# Soluble and cell wall-bound phenolic acids and ferulic acid dehydrodimers in rye flour and five bread model systems: insight into mechanisms of improved availability

**DOI:** 10.1002/jsfa.7007

**Published:** 2014-12-30

**Authors:** Wioletta M Dynkowska, Malgorzata R Cyran, Alicja Ceglińska

**Affiliations:** a Department of Plant Biochemistry and Physiology, Plant Breeding and Acclimatization Institute – National Research InstituteRadzikow, PL-05-870, Blonie, Poland; b Faculty of Food Sciences, Warsaw University of Life SciencesNowoursynowska 159C, PL-02-776, Warsaw, Poland

**Keywords:** rye (*Secale cereale* L.) bread, phenolic acids, ferulic acid dehydrodimers, arabinoxylan-hydrolysing enzymes, feruloyl esterase

## Abstract

**Background:**

The bread-making process influences bread components, including phenolics that significantly contribute to its antioxidant properties. Five bread model systems made from different rye cultivars were investigated to compare their impact on concentration of ethanol-soluble (free and ester-bound) and insoluble phenolics.

**Results:**

Breads produced by a straight dough method without acid addition (A) and three-stage sourdough method with 12 h native starter preparation (C) exhibited the highest, genotype-dependent concentrations of free phenolic acids. Dough acidification by direct acid addition (method B) or by gradual production during prolonged starter fermentation (24 and 48 h, for methods D and E) considerably decreased their level. However, breads B were enriched in soluble ester-bound fraction. Both direct methods, despite substantial differences in dough pH, caused a similar increase in the amount of insoluble ester-bound fraction. The contents of phenolic fractions in rye bread were positively related to activity level of feruloyl esterase and negatively to those of arabinoxylan-hydrolysing enzymes in wholemeal flour.

**Conclusion:**

The solubility of rye bread phenolics may be enhanced by application of a suitable bread-making procedure with respect to rye cultivar, as the mechanisms of this process are also governed by a response of an individual genotype with specific biochemical profile. © 2014 Plant Breeding and Acclimatization Institute, National Research Institute. © 2014 The Authors. *Journal of the Science of Food and Agriculture* published by John Wiley & Sons Ltd on behalf of Society of Chemical Industry.

## Introduction

Among a diverse population of phenolic substances present in cereal grains, phenolic acids (PAs) form a core fraction. They are considered an important grain constituent, mostly owing to their antioxidant activity evidenced by *in vitro* tests.^1^,^2^ Hence, they can limit formation of undesirable oxidation products during grain processing that affect the quality of cereal-based foods as well as their organoleptic characteristics. Once consumed as a diet ingredient, they have the ability to scavenge free radicals and chelate pro-oxidant metals, protecting DNA, lipids and protein from oxidative damage in the human cells that may result in a reduced risk of some chronic diseases, including cancer.^3^–^5^

Ferulic acid (4-hydroxy-3-methoxycinnamic acid) represents 64–88% of the total amount of PAs in cereal grains.^6^ In rye grain, it accounts for 860–1174 µg g^−1^ dry matter (DM).^6^–^9^ A similar level of ferulic acid has been found in wheat grain (772–982 µg g^−1^ DM).^6^,^7^,^10^ The remaining part of minor PAs of cereal grains is composed of sinapic, *p*-coumaric, vanillic, caffeic, syringic and *p*-hyroxybenzoic acids. Unlike fruits and vegetables, cereals contain PAs that occur mostly in an insoluble form bound to dietary fibre (cell wall) components. Thus, their concentration in the bran, being rich in dietary fibre, is two to three times higher than in the wholemeal and four to five times higher than in the endosperm (white) flour.^6^,^7^ Ferulic acid is mainly ester-linked to the *O*-5 position of *α*-l-arabinofuranosyl side substituents on 1 → 4 linked *β*-d-xylopyranosyl residues in the cell wall arabinoxylans.^11^–^13^ However, some amounts of ether-linked ferulic acid have also been detected in seed coat and tissues of crease region of wheat grain.^10^

Cereals comprise an array of dehydrodiferulic acids (DiFAs),^8^,^9^,^12^,^14^ dehydrotriferulic acids^15^–^18^ and dehydrotetraferulic acids,^19^ which are the products of cross-linking of feruloylated cell wall arabinoxylans by radical coupling reaction in the presence of peroxidase and H_2_O_2_ during cell wall development.^20^,^21^ The *in vitro* tests have shown that both forms of the most abundant 8-*O*-4-DiFA, a native form isolated from cereals and which is chemically synthesised, generally exhibit higher antioxidant capacity than ferulic acid.^1^,^14^ Although the antioxidant action of dietary PAs and DiFAs *in vivo* is controlled by multiple factors, a major limiting factor is their availability. It has been demonstrated that the food matrix can be a more important determinant of ferulic acid bioavailability than its metabolism in the intestine and liver.^22^ During processing, the structure and physico-chemical characteristics of the food matrix are changed and the bioaccessibility of its constituents is usually improved.^23^

Rye bread, traditionally consumed in northern and eastern Europe, is an important source of many nutrients, but most of all, dietary fibre and other bioactive components. It is commercially produced from flour with different extraction rates by a variety of bread-making methods, which modify the composition and structure of the bread matrix in a different way. One of the markers of the improved bioavailability of PAs in rye bread is an increased level of their soluble forms. They possess much better solubility in lipid phases than in aqueous ones. However, both types of antioxidant are required to control destructive chain reactions in human cells. There are only a few reports on bread-making-induced changes in rye PAs and DiFAs.^9^,^24^,^25^ They come from the experiments based on one rye cultivar and a certain methodology, in which both principal factors, genetic and technological, cannot be taken into consideration.

The aim of this study was to elucidate how different procedures for making rye bread modify the levels of ethanol-soluble and insoluble PAs as well as DiFAs in the resulting bread. The endosperm (white) and wholemeal flours obtained from five rye cultivars, differing in the content of PAs, have been used to compare the effects of rye cultivar and flour extraction rate on the amount of solubilised PAs in different bread model systems.

## Experimental

### Cereal samples

Rye (*Secale cereale* L.) grain samples, harvested in 2012, were obtained from plant breeding station (DANKO Plant Breeders Ltd, Laski, Poland). Five population cultivars of Polish winter rye (Amilo, Diament, Horyzo, Kier and Stanko), exhibiting a wide range of extract viscosities, were selected. Rye grains were conditioned to 12% moisture at room temperature for 24 h before milling using a Quadrumat Senior mill (Brabender Instruments, Duisburg, Germany). The grains were milled into wholemeal and endosperm flours with extraction rates of 96–98% and 57–64%, respectively.^26^ Each endosperm flour was obtained by standard milling, i.e. break rolling, sifting and reduction rolling, whereas wholemeal was produced by reduction rolling until a similar particle size to that of endosperm flour was achieved, as shown by sieve analysis.

### Rye bread model systems

The straight dough and three-stage sourdough methods were used to prepare a dough from wholemeal and endosperm flours and produce five different breads from each flour. The ingredients and steps of the bread-making procedures are shown in Table1. The rye bread model systems were designed based on the following methods: A, a straight dough method without acid addition; B, a straight dough method with lactic acid addition; C, D and E, the sourdough methods with 12-, 24- and 48-h preparation of a native starter, respectively. The dough composition and parameters of the following steps were modified appropriately to make a comparison between them, when possible.

**Table 1:** Dough ingredients and stages in the preparation of five bread model systems made from rye wholemeal and endosperm flour

Method/stage	Flour (g)	Sourdough starter (g)	Sourdough (g)	Water (mL)	Lactic acid, 50% (mL)	Dry yeast (g)	Salt (g)	Fermentation time^c^ (h)	Proofing time^d^ (h)	Baking time (h)
Straight dough methods										
A	400	—	—	320 (288)[Table-fn tf1-1]	—	1.4	8.0	3	1	0.5
B	400	—	—	314 (282)^a^	5.6	1.4	8.0	3	1	0.5
Sourdough methods										
C/D/E										
One stage – sourdough starter	50	—	—	150 (100)^a^	—	—	—	12/24/48^b^	—	—
Two stage – sourdough	150	150	—	100	—	1.7	—	2.5	—	—
Three stage – dough	300	—	400	160	—	—	10	0.5	1	0.5

a Values in parentheses refer to endosperm flour.

b Time of the first stage (native starter preparation) respectively for methods C, D and E.

c Fermentation time at 30 °C (h).

d Proofing time in pans at 30 °C (h).

The amount of water was adjusted to a dough consistency of 500 BU in a farinograph test on the basis of flour water absorption. Two and three loaves were produced in straight dough and sourdough procedures, respectively. All samples were made in duplicate. The breads were sliced and cut into 1-cm^3^ pieces and immediately freeze-dried.

The grains of winter and spring wheat cultivars (Legenda and Raweta) and freeze-dried breads (commercial samples obtained from local suppliers) were milled in a Cyclotec 1093 laboratory mill (FOSS, Warsaw, Poland) to pass a 0.8 mm sieve and stored in plastic bags with airtight closure at −20 °C. All analyses were carried out at least in duplicate. Moisture was analysed by drying samples for 20 h at 105 °C. For pH measurement, the samples (4 g) were mixed with deionised water (1:6 w/v) and agitated in a shaking water bath for 1 h at 30 °C. The resulting water extract was separated by centrifugation (6000 × *g*, 20 min, room temperature) and its pH value was read with a laboratory pH meter.

### Analysis of ethanol-soluble phenolic acids

The analyses of soluble (free and ester-bound) and insoluble ester-bound PAs are based on a method described by Krygier *et al.*^27^ For extraction of free PAs, the flour and bread samples (300 mg) were accurately weighed in amber Wheaton vials, mixed with 80% ethanol (1 mL) and placed in a shaking water bath at 37 °C for 30 min (under nitrogen). The suspension was centrifuged for 15 min at 3000 × *g*. The supernatant was transferred to a clean amber vial and the residue was extracted with 1 mL of 80% ethanol under the same conditions. The supernatants were combined, mixed with 100 µL of internal standard solution (TMCA, 3,4,5- trimethoxy-(*E*)-cinnamic acid, 10 µg mL^−1^ in 80% ethanol; Sigma–Aldrich, Poznań, Poland) and evaporated under a stream of nitrogen. The dried residue was dissolved in water (2 mL) acidified to pH 2 with 2 mol L^−1^ HCl and extracted with diethyl ether (2 × 2 mL). After centrifugation (3000 × *g*, 15 min), the combined ether phases were dried with anhydrous Na_2_SO_4_ and evaporated to dryness. The dried sample was dissolved in methanol/water (50:50, v/v, 500 µL), filtered through a 0.45 µm syringe filter and transferred to an amber autosampler vial of the high-performance liquid chromatography with diode array detection (HPLC/DAD) system.

The soluble-esterified PAs were extracted twice from flour and bread samples with 80% ethanol, mixed with internal standard solution (TMCA) and evaporated under a stream of nitrogen, as described above. The dried extract residue was saponified with 1 mL of 2 mol L^−1^ NaOH for 4 h at 35 °C with occasional mixing (under nitrogen). The mixture was acidified to pH 2 with 2 mol L^−1^ HCl and extracted with diethyl ether (2 × 2 mL). After centrifugation (3000 × *g*, 10 min), the combined ether phases were dried with anhydrous Na_2_SO_4_ and evaporated to dryness. The dried samples of soluble PAs (free and ester-bound) were dissolved in methanol/water, filtered and analysed using HPLC/DAD. The soluble ester-bound PA concentrations were calculated by difference, i.e. soluble PA content minus soluble free PA content.

### Assessment of ethanol-insoluble phenolic acids and dihydrodiferulic acids

The residue left after ethanol extraction was dried under stream of nitrogen, suspended in 2.9 mL of 2 mol L^−1^ NaOH and hydrolysed for 16 h at 35 °C with occasional mixing. After saponification, the internal standard solution was added (100 µL containing 100 µg of *o*-coumaric acid in 2 mol L^−1^ NaOH, Sigma–Aldrich). The suspension was acidified to pH 2 with 2 mol L^−1^ HCl and extracted with diethyl ether (2 × 3 mL). The combined ether phases were dehydrated with anhydrous Na_2_SO_4_ and evaporated to dryness. The dried samples of ethanol-insoluble phenolics were dissolved in methanol/water, filtered and transferred to an autosampler amber vial, as described above, before HPLC/DAD analysis.

### High-performance liquid chromatography with a diode array detector

Identification and quantification of PAs and DiFAs were performed with a Waters LC system (Waters Associates, Milford, MA, USA) equipped with a photodiode array detector (Waters 996) and a RP-Alltima C_18_ column (250 × 4.6 mm; particles 5 µm in diameter; Alltech Associates Applied Science, Carnforth, UK) maintained at 30 and 45 °C, respectively for ethanol-soluble and insoluble components. A gradient elution program was carried out with mobile phase that consisted of 1 mmol L^−1^ trifluoroacetic acid in water (solution A) and acetonitrile (solution B) as follows: linear gradient from 10% B to 13% B, 0–15 min, isocratic elution 13% B, 15–40 min, gradient elution from 13% B to 50% B, 40–70 min, isocratic elution 50% B, 70–73 min, gradient elution from 50% B to 80% B, 73–74 min, post time, 12 min before the next injection. The flow rate of the mobile phase was 1 mL min^−1^ and the injection volume was 20 µL. The signal of the photodiode array detector was monitored at 280 and 320 nm for quantification of PAs and DiFAs, respectively. They were identified by comparison of their absorption spectra with those previously published.^18^,^28^ The concentrations of the (*Z*)-isomers of caffeic, ferulic, sinapic and *p*-coumaric acids in the samples were quantified using UV-treated and untreated solutions of their (*E*)-isomers. The DiFAs and one dehydrotriferulic acid (TriFA) were quantified using a purified maize bran standard of known, previously published amounts of these components, being a gift from Dr Luc Saulnier (INRA, Nantes, France).^12^,^15^ The PA standards, purchased from Sigma–Aldrich, and maize bran standard were used to prepare the calibration curves, based on an internal standard method and two stock solutions, in 80% ethanol for analysis of free and soluble-bound PAs and in 2 mol L^−1^ NaOH for determination of insoluble PAs and DiFAs. The flour and bread samples of each rye cultivar were analysed in the same run at least in triplicate.

### Assays for enzyme activity

The endo-*β*-d-xylanase, *α*-arabinofuranosidase and *β*-xylosidase activities were assayed in a crude extract, freshly prepared by homogenising flour samples in sodium acetate buffer (1:4 w/v, 10 mmol L^−1^, pH 4.5) as described by Rasmussen *et al.*^29^ Apparent endo-xylanase activity was determined in the extracts using Xylazyme AX tablets as a substrate (Megazyme, Bray, Ireland) according to the procedure described by Cyran *et al.*,^30^ and expressed in EU g^−1^ DM. One EU is the amount of enzyme needed to increase the extinction at 590 nm by 1.0, under the conditions of the assay, in 2 h. The *α*-arabinofuranosidase and *β*-xylosidase activities were measured in the crude extracts by using *p*-nitrophenyl-*β*-d-xylopyranoside and *p*-nitrophenyl-*α*-l-arabinofuranoside (Sigma–Aldrich).^29^ The activities of *α*-arabinofuranosidase and *β*-xylosidase were expressed in pkatal g^−1^ of flour dry mass, where 1 pkatal produces 1 pmol product s^−1^ at the pH and temperature of incubation.

Ferulic acid esterase activity was measured in a crude extract prepared by homogenising flour samples in phosphate buffer (1:10 w/v, 80 mmol L^−1^ M, pH 7.0), using ethyl ferulate (ethyl 4-hydroxy-3-methoxycinnamate; Sigma–Aldrich) as a substrate. The procedure used followed that described by Hansen *et al.*^9^ with some modifications. The amount of ferulic acid released from substrate during incubation with the crude extracts was quantified with the HPLC/DAD system, as described above, using TMCA as internal standard. The blanks made up of crude extract or substrate combined with phosphate buffer were included in analysis of each crude extract to correct for the presence of free ferulic acid in the crude extract and to check the stability of ethyl ferulate under incubation conditions. The activity of ferulic acid esterase was expressed as pkatal g^−1^ of dry matter. All enzyme activity assays were performed in triplicate on each crude extract.

### Statistical analysis

The data obtained for different cultivars and bread-making methodologies were subjected to a multiple linear regression using the STATISTICA software (StatSoft, Kraków, Poland). To measure the relative importance of each independent variable present in a regression model, the relative weight analysis was applied, as described previously.^31^,^32^ Additionally, Pearson correlation coefficients (*r*) were calculated between the analysed parameters across the bread model systems.

## Results and Discussion

### Analytical methodology

**Figure 1:**
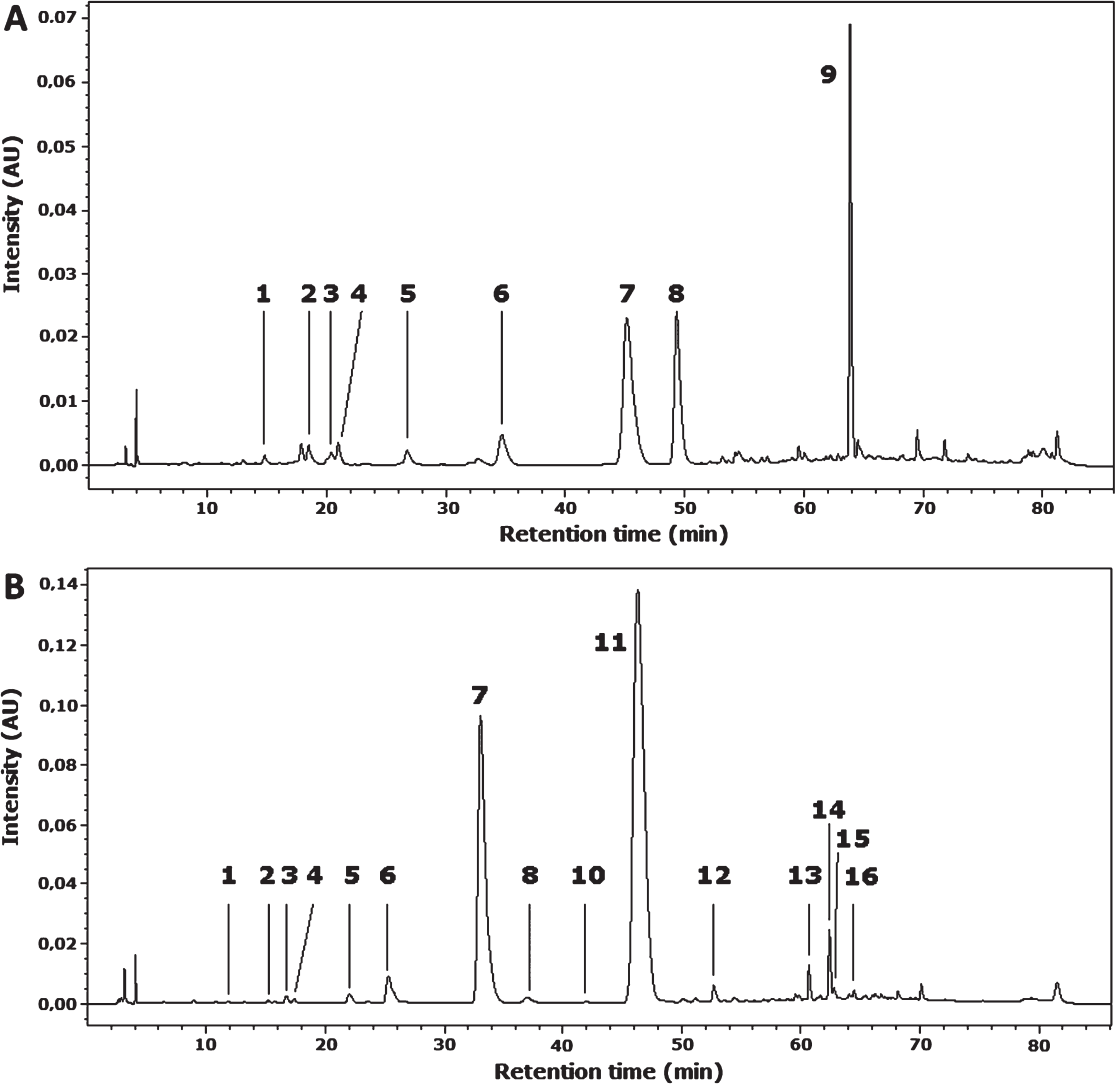
HPLC elution profiles of (A) ethanol-soluble (free and ester-bound) PAs separated on a column at 30 °C, and (B) insoluble ester-bound phenolics separated on column at 45 °C isolated from rye wholemeal (cv. Horyzo), detection at 280 nm: (1) *p*-hydroxybenzoic acid, (2) vanillic acid, (3) (*E*)-caffeic acid, (4) syringic acid, (5) vanillin, (6) (*E*)-*p*-coumaric acid, (7) (*E*)-ferulic acid, (8) (*E*)-sinapic acid (9) TMCA, internal standard, (10) 8-8′-DiFA aryltetralin, (11) *o*-coumaric acid, internal standard, (12) 8-5′-DiFA benzofuran form, (13) 5-5′-DiFA, (14) 8-*O*-4′-DiFA, (15) 8-5′-DiFA, (16) 4-O-8′, 5′-5″-TriFA.

An analytical protocol used in this study was optimised in a preliminary experiment with respect to yield of analysed PAs and minimisation of UV-induced changes of their native forms. As can be seen in Fig. 1, the HPLC chromatograms contained only (*E*)-isomers of hydroxycinnamic acids, indicating that conditions applied effectively limited formation of their (*Z*) forms. Although higher extraction temperature ensures better extraction efficiency, the ethanol-soluble components were extracted at the temperature of the human body, bearing in mind that bread constitutes an element of the human diet. The preliminary experiment showed that 1, 2 and 4 h of saponification of ethanol-insoluble residue from rye wholemeal in 2 mol L^−1^ NaOH at 37 °C resulted in 70, 73 and 79% recovery of ferulic acid, in relation to that obtained after 16 h of saponification. For sinapic acid these values were 74, 81 and 87%, and for *p*-coumaric acid 54, 63 and 71%, respectively. It is known that cell walls are embedded in the starch–protein matrix of cereal grain, which creates a physical barrier and, thus, alkaline de-esterification of cell wall polymers in rye flour and bread is a time-consuming process. To release ferulic acid from ethanol-soluble esters, 4 h of saponification was necessary, whereas sinapic acid was completely released after 2 h.

### Ethanol-soluble phenolic acids

**Figure 2:**
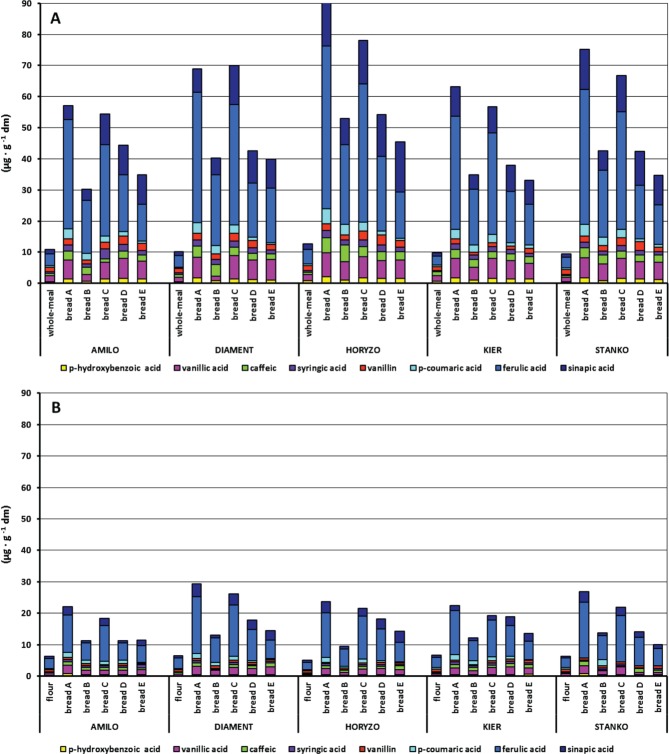
Content and composition of soluble free PAs in (A) rye wholemeals and (B) endosperm flours and resulting breads from five experimental model systems. PAs are arranged from *p*-hydroxybenzoic acid to sinapic acid from the bottom of each column to the top.

The concentration of free PAs ranged from 10 to13 µg g^−1^ DM in rye wholemeals, and from 5 to 7 µg g^−1^ DM in endosperm flours (Fig. 2), which accounted for 1% and 2–3% of total PAs, respectively. Free PAs of rye wholemeal were composed of ferulic acid (31–38%), vanillic acid (15–19%), vanillin (13–18%), sinapic acid (10–14%), caffeic acid (5–12%), *p*-hydroxybenzoic acid (5–6%) and syringic acid (4–5%), whereas those of endosperm flour were characterised by a higher proportion of ferulic acid (46–51%). The level of free ferulic acid found in rye wholemeals (3.0–4.5 µg g^−1^ DM) was similar to that reported by Hansen *et al.*^9^ (3.3 µg g^−1^ DM). In the endosperm flours, its content varied from 2.3 to 3.1 µg g^−1^ DM.

All types of rye bread were enriched in free PAs, in comparison to starting flours, and similar trends were observed for wholemeal and endosperm breads across five rye cultivars (Fig. 2). Clearly, their amounts in the breads were dependent on both cultivar and bread-making method used. Among the five procedures, two of them, A and C, provided the most favourable conditions for solubilisation of PAs. Breads A, obtained by a direct method without acid addition, contained five to eight times more free PAs (57–94 µg g^−1^ DM) than in the starting wholemeals and three to five times more (22–29 µg g^−1^ DM) than in the endosperm flours. A similar or slightly lower concentration of free PAs was observed in breads C, produced by the sourdough method with the shortest (12 h) fermentation of the native starter (54–78 and 19–26 µg g^−1^ DM).

The rye breads displayed much lower pH values than did corresponding flours (Table2). The pH values of breads A and C, for an individual rye cultivar, were close to each other. However, they were much higher than those of breads B and E, obtained by the direct method with lactic acid addition, and the sourdough method with prolonged 48 h fermentation of the starter. Breads B with the lowest pH values had markedly lower levels of free PAs (30–53 and 10–14 µg g^−1^ DM), when compared with those of breads A and C. Considering that changes in the pH values of rye breads are related to those of corresponding doughs, a negative effect of their low pH on the solubilisation of free PAs during dough fermentation was well shown. Nevertheless, a relatively small decrease in pH values between sourdough breads C and D, obtained after 12-h and 24-h starter fermentations, generally yielded a significant drop in the level of free PAs in breads D, reaching that found in breads B with the lowest pH. This may indicate their conversion by native microorganisms during sourdough fermentation. Lactic acid bacteria are the principal microorganisms in the sourdough, which co-exist with yeasts. It has been shown that *p*-coumaric, caffeic and ferulic acids are metabolised to their ethyl and vinyl derivatives by *Lactobacillus plantarum*, the most abundant lactic acid bacterial species involved in fermentation process of plant materials.^33^ Also, yeasts have the ability to produce ferulic acid decarboxylase and metabolise ferulic acid to 4-vinyl guaiacol.^34^ Such transformation of free PAs could take place during fermentation of doughs E as well, since their amounts found in these breads (33–45 and 11–15 µg g^−1^ DM) were lower or comparable to those of breads D.

**Table 2:** Content (µg g^−1^ dm) of ethanol-soluble ester-bound phenolic acids (PAs) in rye wholemeals, endosperm flours and resulting breads in comparison to commercial samples and activities of endo-xylanase, *α*-arabinofuranosidase, *β*-xylosidase and feruloyl esterase in starting flours

Sample	Flour/bread	pH	*p*-Coumaric acid	Ferulic acid	Sinapic acid	Total ester-bound PAs^a^	Endo-xylanase (UE g^−1^ dm)	*α*-Arabinofuranosidase (pkatal g^−1^ dm)	*β*-Xylosidase (pkatal g^−1^ dm)	Feruloyl esterase (pkatal g^−1^ dm)
Amilo	Wholemeal	6.63	3.6 ± 0.1	45 ± 1	97 ± 5	159 ± 6 (169)^b^	0.252 ± 0.001	834 ± 14	682 ± 2	178 ± 10
	Bread A	6.15	3.4 ± 0.0	40 ± 1	88 ± 1	141 ± 2 (195)	—	—	—	—
	Bread B	4.54	3.9 ± 0.1	42 ± 1	92 ± 1	152 ± 2 (180)	—	—	—	—
	Bread C	6.18	3.8 ± 0.1	39 ± 0	76 ± 3	128 ± 4 (179)	—	—	—	—
	Bread D	6.14	3.7 ± 0.1	39 ± 1	87 ± 1	138 ± 1 (178)	—	—	—	—
	Bread E	5.84	3.4 ± 0.0	41 ± 2	95 ± 4	146 ± 7 (178)	—	—	—	—
Diament	Wholemeal	6.65	3.8 ± 0.1	47 ± 4	87 ± 6	151 ± 6 (160)	0.239 ± 0.001	939 ± 9	719 ± 3	206 ± 3
	Bread A	6.16	4.2 ± 0.2	49 ± 3	99 ± 1	164 ± 3 (231)	—	—	—	—
	Bread B	4.67	4.6 ± 0.1	48 ± 2	102 ± 1	168 ± 4 (208)	—	—	—	—
	Bread C	6.22	4.2 ± 0.3	45 ± 2	86 ± 1	146 ± 3 (216)	—	—	—	—
	Bread D	6.13	3.6 ± 0.0	40 ± 1	77 ± 3	130 ± 1 (172)	—	—	—	—
	Bread E	5.60	4.1 ± 0.2	39 ± 1	76 ± 4	128 ± 1 (168)	—	—	—	—
Horyzo	Wholemeal	6.68	4.9 ± 0.0	58 ± 3	111 ± 1	183 ± 3 (196)	0.220 ± 0.001	750 ± 4	649 ± 3	196 ± 6
	Bread A	6.18	4.4 ± 0.2	49 ± 4	104 ± 1	167 ± 4 (260)	—	—	—	—
	Bread B	4.71	5.4 ± 0.3	73 ± 7	119 ± 2	210 ± 8 (263)	—	—	—	—
	Bread C	6.23	4.8 ± 0.1	50 ± 5	93 ± 3	159 ± 5 (237)	—	—	—	—
	Bread D	6.16	4.8 ± 0.0	46 ± 1	92 ± 1	154 ± 2 (209)	—	—	—	—
	Bread E	5.84	4.4 ± 0.2	48 ± 4	101 ± 2	164 ± 1 (207)	—	—	—	—
Kier	Wholemeal	6.62	4.2 ± 0.2	43 ± 1	71 ± 5	130 ± 6 (138)	0.324 ± 0.005	970 ± 8	746 ± 12	187 ± 14
	Bread A	6.15	4.5 ± 0.3	41 ± 3	70 ± 6	128 ± 5 (188)	—	—	—	—
	Bread B	4.61	4.8 ± 0.1	44 ± 2	68 ± 7	130 ± 8 (164)	—	—	—	—
	Bread C	6.20	4.9 ± 0.0	42 ± 0	70 ± 1	131 ± 2 (184)	—	—	—	—
	Bread D	6.17	5.0 ± 0.2	40 ± 0	68 ± 1	127 ± 1 (161)	—	—	—	—
	Bread E	5.41	4.0 ± 0.2	38 ± 0	60 ± 2	113 ± 1 (110)	—	—	—	—
Stanko	Wholemeal	6.58	4.3 ± 0.2	46 ± 1	80 ± 6	142 ± 7 (151)	0.258 ± 0.002	922 ± 7	796 ± 10	193 ± 12
	Bread A	6.11	4.5 ± 0.4	44 ± 2	83 ± 1	143 ± 3 (213)	—	—	—	—
	Bread B	4.68	5.2 ± 0.2	50 ± 0	99 ± 1	167 ± 1 (206)	—	—	—	—
	Bread C	6.20	5.2 ± 0.2	48 ± 2	83 ± 1	147 ± 1 (214)	—	—	—	—
	Bread D	6.17	5.0 ± 0.0	45 ± 2	86 ± 3	148 ± 5 (190)	—	—	—	—
	Bread E	5.38	4.4 ± 0.0	43 ± 3	83 ± 4	139 ± 8 (172)	—	—	—	—
Amilo	Endosperm flour (57%)^c^	6.49	1.0 ± 0.2	15 ± 0	14 ± 0	34 ± 0 (41)^b^	0.092 ± 0.001	319 ± 4	382 ± 8	49 ± 2
	Bread A	6.02	0.7 ± 0.0	13 ± 0	16 ± 0	31 ± 0 (53)	—	—	—	—
	Bread B	3.94	0.8 ± 0.1	14 ± 1	14 ± 1	31 ± 3 (43)	—	—	—	—
	Bread C	6.03	0.7 ± 0.1	13 ± 0	13 ± 1	29 ± 2 (48)	—	—	—	—
	Bread D	6.02	0.8 ± 0.5	14 ± 1	14 ± 2	31 ± 2 (43)	—	—	—	—
	Bread E	5.91	0.7 ± 0.1	12 ± 0	13 ± 1	26 ± 2 (38)	—	—	—	—
Diament	Endosperm flour (64%)	6.52	1.5 ± 0.1	24 ± 2	24 ± 1	56 ± 0 (63)	0.151 ± 0.002	421 ± 8	492 ± 3	77 ± 11
Bread A	6.02	1.3 ± 0.0	19 ± 1	20 ± 0	45 ± 0 (74)	—	—	—	—	
Bread B	4.03	1.2 ± 0.1	20 ± 0	24 ± 2	51 ± 2 (64)	—	—	—	—	
Bread C	6.03	1.0 ± 0.2	20 ± 1	21 ± 1	47 ± 2 (74)	—	—	—	—	
Bread D	6.00	1.3 ± 0.1	22 ± 2	24 ± 2	52 ± 2 (70)	—	—	—	—	
Bread E	5.95	1.1 ± 0.1	17 ± 0	19 ± 1	40 ± 1 (54)	—	—	—	—	
Horyzo	Endosperm flour (63%)	6.49	1.5 ± 0.1	24 ± 0	31 ± 1	61 ± 1 (66)	0.106 ± 0.002	367 ± 12	399 ± 9	65 ± 8
	Bread A	6.00	1.3 ± 0.1	18 ± 1	18 ± 1	41 ± 2 (65)	—	—	—	—
	Bread B	3.98	1.7 ± 0.0	19 ± 0	22 ± 0	48 ± 0 (58)	—	—	—	—
	Bread C	6.08	1.4 ± 0.1	19 ± 1	20 ± 1	45 ± 3 (67)	—	—	—	—
	Bread D	6.01	1.2 ± 0.1	18 ± 1	22 ± 0	45 ± 1 (63)	—	—	—	—
	Bread E	5.94	1.1 ± 0.1	17 ± 0	19 ± 0	40 ± 0 (55)	—	—	—	—
Kier	Endosperm flour (63%)	6.51	1.3 ± 0.1	20 ± 0	29 ± 1	55 ± 1 (61)	0.141 ± 0.004	363 ± 3	397 ± 9	54 ± 2
	Bread A	6.03	1.4 ± 0.0	17 ± 1	21 ± 0	43 ± 0 (65)	—	—	—	—
	Bread B	4.03	1.3 ± 0.0	16 ± 0	21 ± 1	43 ± 0 (55)	—	—	—	—
	Bread C	6.03	1.4 ± 0.2	15 ± 0	19 ± 0	39 ± 1 (58)	—	—	—	—
	Bread D	6.01	1.2 ± 0.0	18 ± 1	22 ± 0	45 ± 1 (64)	—	—	—	—
	Bread E	5.56	1.3 ± 0.0	17 ± 1	22 ± 0	43 ± 1 (56)	—	—	—	—
Stanko	Endosperm flour (64%)	6.49	1.4 ± 0.0	22 ± 2	18 ± 0	46 ± 2 (52)	0.108 ± 0.003	405 ± 15	520 ± 8	71 ± 10
	Bread A	5.97	1.7 ± 0.1	20 ± 0	20 ± 0	45 ± 0 (73)	—	—	—	—
	Bread B	4.03	0.6 ± 0.1	21 ± 1	23 ± 0	53 ± 0 (67)	—	—	—	—
	Bread C	6.01	1.5 ± 0.3	20 ± 1	20 ± 1	45 ± 2 (68)	—	—	—	—
	Bread D	6.00	0.9 ± 0.0	19 ± 0	21 ± 1	47 ± 1 (62)	—	—	—	—
	Bread E	5.77	0.5 ± 0.1	19 ± 0	21 ± 1	46 ± 0 (57)	—	—	—	—
Legenda (winter wheat)		—	0.7 ± 0.1	12 ± 1	16 ± 0	42 ± 1 (47)	—	—	—	—
Raweta (spring wheat)		—	1.5 ± 0.2	19 ± 0	42 ± 1	71 ± 1 (74)	—	—	—	—
White wheat bread		—	0.8 ± 0.0	12 ± 3	15 ± 0	31 ± 4 (35)	—	—	—	—
Wheat crisp bread		—	0.8 ± 0.1	14 ± 0	30 ± 0	52 ± 0 (59)	—	—	—	—
Rye crisp bread		—	2.5 ± 0.2	24 ± 2	47 ± 3	77 ± 6 (94)	—	—	—	—
Pumpernickel bread		—	5.1 ± 0.2	37 ± 1	56 ± 0	106 ± 1 (133)	—	—	—	—

Data represent mean ± standard deviation.

a Sum of *p*-hydroxybenzoic acid, vanillic acid, caffeic acid, syringic acid, vanillin, *p*-coumaric acid, sinapic acid and ferulic acid.

b Values in parentheses refer to sum of soluble (free and ester-bound) PAs.

c Flour extraction rate.

Rye wholemeal breads contained 12–52 µg g^−1^ DM free ferulic acid, 4–16 µg g^−1^ DM sinapic acid and 2–8 µg g^−1^ DM vanillic acid (Fig. 2). The remaining PAs identified (caffeic, *p*-coumaric and *p*-hydroxybenzoic acids) and vanillin were the minor components. These results are consistent with the study by Rosén *et al.*^35^ who reported free ferulic, sinapic and vanillic acids contents of 26–32 µg g^−1^ DM, 9–11 µg g^−1^ DM and 8–11 µg g^−1^ DM, respectively, in wholemeal breads produced by a direct method without acid addition. A similar proportion of ferulic (5–18 µg g^−1^ DM), sinapic (1–4 µg g^−1^ DM) and vanillic (1–3 µg g^−1^ DM) acids was found in rye breads made from endosperm flour (Fig. 2).

Esterified ethanol-soluble PAs accounted for 89–94% and 85–92% of total soluble fraction and 7–8% and 16–19% of total PAs in rye wholemeal and endosperm flour, respectively. Their contents ranged from 130 to 183 µg g^−1^ DM in wholemeals and from 34 to 61 µg g^−1^ DM in endosperm flours (Table2). The differences in the levels of these components between flour and breads produced by different bread-making procedures were relatively small. There was no clear relationship between soluble ester-bound PAs present in the wholemeal flour and bread, while the endosperm breads, in most cases, had lower content of that fraction, in comparison to starting flour. However, its concentration in the resulting bread was dependent on rye cultivar used for bread-making as well as flour extraction rate, in the case of endosperm flour. This may indicate that soluble esters of PAs present in the endosperm flour constituted a major substrate for hydrolytic release of their free form, unlike the corresponding wholemeal fraction.

The profiles of soluble PAs esterified to oligomers of rye wholemeals were different from those observed in endosperm flours, although the flours and resulting breads had similar compositions. Among soluble ester-bound PAs, sinapic acid predominated in wholemeal flours and breads, representing 52–65% of this fraction. The concentration of ferulic acid was almost two times lower (27–35%), whereas *p*-coumaric acid constituted only 2–4% of total ester-bound PAs. In contrast, the endosperm flours and breads contained similar amounts of sinapic and ferulic acids, which constituted 42–53% and 36–47% of their total fraction. This indicates the differences in the distribution of both soluble ester-bound PAs within rye grain.

In all wholemeal breads, the levels of soluble PAs (sum of free and ester-bound counterparts) were increased when compared to those of starting flours (Table2). The breads produced by methods A and C displayed the highest increase of this fraction (33–44% and 21–42%, respectively), excluding those from Amilo (15% for bread A and 5–6% for the remaining breads). This was mostly due to considerable changes in the content of free PAs found in these breads. However, the wholemeal breads B, obtained by the direct method with lactic acid addition from Diament, Horyzo and Stanko, also showed markedly increased levels of the soluble fraction (30–37%), being a result of their greater concentration of soluble ester-bound counterparts, while only 18% and 6% increases were noted for breads B made from rye cultivars Kier and Amilo. The sourdough methods D and E with extended 24- and 48-h fermentation of native starters, in the most cases, resulted in much smaller increases of soluble fraction in the breads. The endosperm breads A and C were also enriched in soluble PAs, except for those obtained from Horyzo and Kier, where a decline in the levels of this fraction was observed.

### Activity of endogenous enzymes

The apparent endo-xylanase activity levels in wholemeals and endosperm flours of five rye cultivars are summarised in Table2. They were in line with those previously reported for wholemeal and endosperm flour of rye cultivars grown in 2008 at the same location.^36^ The *α*-arabinofuranosidase activity levels were somewhat higher than those of *β*-xylosidase in the wholemeals. An opposite relationship was observed in endosperm flour; however, their values were approximately two times lower. Hansen *et al.*^9^ reported comparable activities of these enzymes (896 and 862 pkatal g^−1^ DM) for rye wholemeal.

The apparent activity level of feruloyl esterase varied from 178 to 206 pkatal g^−1^ DM in wholemeals and from 49 to 77 pkatal g^−1^ DM in endosperm flours. A similar value was previously reported for one rye wholemeal (219 pkatal g^−1^ DM).^9^ The wholemeal activity of feruloyl esterase was significantly correlated with free ferulic acid content in sourdough bread E (*r* = 0.92, *P* < 0.05). The lower correlation coefficients were found for breads A, B and C (*r* = 0.59, *r* = 0.77 and *r* = 0.75, respectively). Moreover, it showed a relationship with the pH value of breads B and C (*r* = 0.81 and *r* = 0.84), while the activities of endo-xylanase, *α*-arabinofuranosidase and *β*-xylosidase were negatively associated with concentrations of free PAs (*r* = −0.90, *r* = −0.94 and *r* = −0.91, *P* < 0.05, respectively) and soluble ester-bound PAs (*r* = −0.86, *r* = −0.95, *P* < 0.05, and *r* = −0.84) in wholemeal and similar trends were also observed for both PA fractions in the resulting breads.

In contrast, the activities of *α*-arabinofuranosidase and *β*-xylosidase in endosperm flours were positively related to the concentration of free ferulic acid in the endosperm breads A, B and C (*r* = 0.98, *r* = 0.88 and *r* = 0.93 for *α*-arabinofuranosidase, and *r* = 0.95, *r* = 0.92 and *r* = 0.83 for *β*-xylosidase). Also, the activity of feruloyl esterase of endosperm flour was positively correlated with the amount of free ferulic acid in the breads A and C (*r* = 0.94 and *r* = 0.97, *P* < 0.05) and lower correlation coefficients were observed for breads B and D (*r* = 0.76 and *r* = 0.63).

### Ethanol-insoluble phenolic acids and dihydrodiferulic acids

The PAs released by saponification from the residue left after ethanol extraction represent fraction that is ester-linked to the cell-wall components, mainly arabinoxylans, commonly classified as water-extractable and unextractable polysaccharides. As 80% ethanol is used for precipitation of their high-molecular-weight fraction from aqueous extracts in routine analysis of these polysaccharides, the ethanol-insoluble PAs evaluated in this study are ester-linked to both arabinoxylan fractions and other cell-wall components, especially lignin.^37^ Nevertheless, some amounts of feruloylated arabinoxylan oligosaccharides, being the products of their partial depolymerisation during bread-making, may be recovered in ethanol-soluble fraction of the bread.

The direct bread-making methods A and B generally resulted in increased concentration of insoluble-bound PAs in the wholemeal bread when compared to that of starting flour (Table3). In wholemeal bread A their content was increased by 10–17% and only by 4% for that made from Diament. An increase of 15–18% was found in wholemeal bread B, excluding that from Diament, where practically no change was found.

**Table 3:** Content (µg g^−1^ dm) of ethanol-insoluble phenolic acids, dehydrodimers (DiFAs) and dehydrotrimer (TriFA) of ferulic acid in rye wholemeals, endosperm flours and resulting breads in comparison to commercial samples

Sample	Flour/bread	*p*-Coumaric acid	Ferulic acid	Sinapic acid	Total insoluble PAs^a^	8,8′-DiFA aryltetralin form	5,5′-DiFA	8-*O*-4′-DiFA	8,5′-DiFA	TriFA^b^	8,5′-DiFA benzofuran form	Total identified DiFAs
Amilo	Wholemeal	39 ± 0	921 ± 15	84 ± 3	1072 ± 19	5.3 ± 0.4	46 ± 1	125 ± 1	32 ± 2	30 ± 6	66 ± 0	303 ± 10
	A bread	43 ± 3	1010 ± 17	96 ± 2	1181 ± 22	9.1 ± 0.6	50 ± 2	138 ± 6	24 ± 1	38 ± 1	60 ± 2	319 ± 11
	B bread	44 ± 0	1070 ± 17	106 ± 1	1250 ± 18	9.2 ± 1.5	46 ± 0	126 ± 0	22 ± 0	24 ± 1	61 ± 0	289 ± 0
	C bread	36 ± 0	878 ± 2	79 ± 1	1020 ± 3	6.8 ± 0.0	41 ± 2	110 ± 5	21 ± 0	21 ± 4	52 ± 1	252 ± 12
	D bread	36 ± 1	885 ± 17	81 ± 1	1030 ± 17	5.6 ± 1.4	40 ± 1	112 ± 3	17 ± 1	22 ± 3	55 ± 1	253 ± 3
	E bread	38 ± 1	946 ± 23	88 ± 8	1101 ± 23	7.4 ± 0.3	44 ± 0	122 ± 4	21 ± 1	24 ± 1	57 ± 1	272 ± 5
Diament	Wholemeal	53 ± 5	1033 ± 5	79 ± 2	1204 ± 3	6.0 ± 0.3	50 ± 3	146 ± 1	37 ± 1	40 ± 3	75 ± 3	354 ± 11
	A bread	50 ± 0	1072 ± 3	93 ± 1	1250 ± 2	7.9 ± 0.8	55 ± 0	155 ± 3	29 ± 1	42 ± 5	68 ± 2	356 ± 6
	B bread	48 ± 0	1032 ± 6	87 ± 0	1201 ± 9	9.5 ± 1.1	53 ± 3	149 ± 1	29 ± 3	42 ± 2	65 ± 0	347 ± 8
	C bread	49 ± 1	1023 ± 11	86 ± 2	1196 ± 16	11.4 ± 2.1	52 ± 2	148 ± 7	27 ± 3	51 ± 4	66 ± 2	355 ± 16
	D bread	44 ± 1	951 ± 5	76 ± 2	1104 ± 8	10.1 ± 0.1	47 ± 2	134 ± 0	23 ± 0	39 ± 1	61 ± 0	314 ± 0
	E bread	44 ± 1	963 ± 3	73 ± 4	1115 ± 5	10.4 ± 0.4	48 ± 3	137 ± 2	24 ± 2	36 ± 4	61 ± 2	316 ± 13
Horyzo	Wholemeal	46 ± 1	932 ± 16	80 ± 2	1090 ± 19	5.9 ± 0.2	43 ± 3	126 ± 5	32 ± 0	27 ± 1	67 ± 1	301 ± 10
	A bread	56 ± 0	1083 ± 9	103 ± 1	1276 ± 9	7.6 ± 0.1	50 ± 2	144 ± 4	28 ± 3	36 ± 3	68 ± 1	333 ± 11
	B bread	55 ± 1	1089 ± 13	101 ± 1	1281 ± 26	7.8 ± 0.1	51 ± 2	144 ± 3	32 ± 1	37 ± 4	67 ± 0	338 ± 10
	C bread	54 ± 1	1066 ± 14	103 ± 1	1256 ± 17	7.7 ± 0.0	49 ± 0	142 ± 2	26 ± 1	35 ± 2	68 ± 2	328 ± 4
	D bread	52 ± 1	1077 ± 8	101 ± 0	1262 ± 8	7.1 ± 0.2	48 ± 2	139 ± 1	28 ± 1	36 ± 4	65 ± 0	322 ± 8
	E bread	54 ± 1	1067 ± 18	99 ± 3	1254 ± 26	7.4 ± 0.4	49 ± 1	141 ± 6	27 ± 2	36 ± 3	66 ± 0	326 ± 11
Kier	Wholemeal	44 ± 1	850 ± 15	56 ± 4	984 ± 14	4.5 ± 0.3	39 ± 4	114 ± 4	30 ± 1	25 ± 2	64 ± 2	276 ± 12
	A bread	51 ± 0	978 ± 13	81 ± 5	1143 ± 20	6.6 ± 0.3	43 ± 1	124 ± 3	23 ± 0	25 ± 3	63 ± 0	284 ± 7
	B bread	50 ± 1	968 ± 15	75 ± 4	1131 ± 21	7.1 ± 0.4	47 ± 1	135 ± 3	31 ± 1	38 ± 3	63 ± 1	321 ± 9
	C bread	50 ± 1	987 ± 4	81 ± 2	1152 ± 5	5.9 ± 0.1	46 ± 5	133 ± 8	26 ± 3	34 ± 6	66 ± 0	311 ± 13
	D bread	50 ± 1	957 ± 6	78 ± 2	1116 ± 7	7.1 ± 0.2	49 ± 1	138 ± 0	26 ± 1	42 ± 0	66 ± 0	328 ± 0
	E bread	46 ± 0	891 ± 11	68 ± 4	1034 ± 17	7.0 ± 0.0	45 ± 2	128 ± 5	26 ± 1	35 ± 1	61 ± 2	301 ± 9
Stanko	Wholemeal	45 ± 3	907 ± 19	82 ± 5	1064 ± 22	6.2 ± 0.1	39 ± 2	112 ± 8	27 ± 2	21 ± 3	63 ± 4	269 ± 11
	A bread	48 ± 1	1015 ± 17	92 ± 1	1187 ± 16	6.8 ± 0.7	41 ± 2	116 ± 6	19 ± 1	22 ± 4	61 ± 2	265 ± 15
	B bread	57 ± 5	1065 ± 2	92 ± 3	1247 ± 5	8.8 ± 0.3	49 ± 3	134 ± 6	25 ± 1	25 ± 1	66 ± 2	307 ± 10
	C bread	47 ± 1	918 ± 9	88 ± 2	1082 ± 10	7.0 ± 0.6	42 ± 4	114 ± 4	19 ± 3	24 ± 6	60 ± 2	266 ± 9
	D bread	47 ± 4	949 ± 15	90 ± 5	1115 ± 21	6.3 ± 0.1	43 ± 3	121 ± 5	21 ± 1	26 ± 3	60 ± 3	277 ± 8
	E bread	52 ± 1	1040 ± 2	102 ± 1	1225 ± 2	8.1 ± 0.9	47 ± 2	129 ± 6	23 ± 1	24 ± 3	63 ± 1	294 ± 7
Amilo	Endosperm flour	6.1 ± 0.6	137 ± 8	2.2 ± 0.2	152 ± 9	0.8 ± 0.3	4.6 ± 0.5	15 ± 2	3.1 ± 0.2	2.2 ± 0.2	8.7 ± 0.2	35 ± 4
	A bread	7.4 ± 0.5	181 ± 7	8.7 ± 0.4	207 ± 6	1.4 ± 0.2	6.5 ± 0.4	18 ± 4	3.0 ± 0.1	2.9 ± 0.1	10.1 ± 0.1	42 ± 4
	B bread	7.6 ± 0.1	179 ± 5	9.2 ± 0.0	206 ± 5	2.2 ± 0.0	6.5 ± 0.3	20 ± 2	3.3 ± 0.0	3.3 ± 0.1	10.7 ± 0.4	46 ± 3
	C bread	7.6 ± 0.4	174 ± 1	9.1 ± 0.5	199 ± 3	1.5 ± 0.1	6.4 ± 0.3	18 ± 1	2.3 ± 0.0	2.9 ± 0.4	10.3 ± 0.2	41 ± 1
	D bread	6.8 ± 0.0	165 ± 2	9.9 ± 0.4	188 ± 3	1.9 ± 0.1	6.3 ± 0.4	18 ± 2	2.9 ± 0.3	2.8 ± 0.4	10.5 ± 0.3	43 ± 3
	E bread	6.7 ± 0.8	171 ± 5	8.6 ± 0.7	192 ± 6	1.9 ± 0.1	6.4 ± 0.4	20 ± 1	2.8 ± 0.2	2.7 ± 0.1	10.7 ± 0.3	44 ± 2
Diament	Endosperm flour	9.1 ± 0.4	224 ± 8	4.3 ± 0.5	248 ± 10	1.4 ± 0.0	9.9 ± 0.3	33 ± 4	6.3 ± 0.3	5.4 ± 0.5	17.2 ± 0.2	73 ± 7
	A bread	9.5 ± 0.2	240 ± 6	15.4 ± 0.4	275 ± 6	2.0 ± 0.4	11.9 ± 0.5	40 ± 2	5.8 ± 0.6	7.3 ± 0.2	17.2 ± 0.4	84 ± 5
	B bread	9.6 ± 0.3	243 ± 6	13.6 ± 0.8	276 ± 11	2.5 ± 0.0	11.8 ± 0.8	40 ± 2	6.5 ± 0.4	7.8 ± 0.1	18.0 ± 0.5	87 ± 5
	C bread	9.4 ± 0.2	283 ± 7	11.3 ± 0.9	313 ± 12	2.2 ± 0.3	11.9 ± 0.5	40 ± 1	5.7 ± 0.1	7.9 ± 0.5	17.8 ± 0.4	85 ± 2
	D bread	8.7 ± 0.1	222 ± 3	10.7 ± 0.5	249 ± 8	1.9 ± 0.0	10.9 ± 0.2	37 ± 1	5.4 ± 0.1	7.2 ± 0.4	16.4 ± 0.0	77 ± 2
	E bread	9.2 ± 0.0	226 ± 5	13.6 ± 0.8	257 ± 4	1.9 ± 0.2	11.6 ± 0.3	38 ± 0	5.1 ± 0.3	8.0 ± 0.5	16.8 ± 0.2	81 ± 2
Horyzo	Endosperm flour	9.4 ± 0.6	217 ± 9	8.7 ± 0.4	244 ± 11	1.3 ± 0.1	8.6 ± 0.4	30 ± 3	7.0 ± 0.5	5.1 ± 0.3	15.3 ± 0.2	67 ± 5
	A bread	10.2 ± 0.2	214 ± 3	17.2 ± 0.5	251 ± 3	1.9 ± 0.2	9.5 ± 0.1	31 ± 1	4.8 ± 0.2	5.7 ± 0.3	14.0 ± 0.5	67 ± 1
	B bread	9.5 ± 0.4	208 ± 4	15.8 ± 0.5	242 ± 6	2.2 ± 0.0	9.2 ± 0.5	31 ± 2	5.5 ± 0.2	5.4 ± 0.5	14.2 ± 0.9	67 ± 5
	C bread	9.8 ± 0.1	210 ± 0	15.0 ± 0.1	244 ± 0	1.5 ± 0.0	9.5 ± 0.2	31 ± 1	4.2 ± 0.1	4.8 ± 0.1	14.2 ± 0.1	65 ± 2
	D bread	9.8 ± 0.7	229 ± 3	16.9 ± 0.2	265 ± 3	2.3 ± 0.4	10.7 ± 0.3	35 ± 0	5.0 ± 0.0	6.1 ± 0.2	15.6 ± 0.5	75 ± 1
	E bread	9.6 ± 0.0	217 ± 5	16.3 ± 0.4	252 ± 7	2.4 ± 0.1	10.2 ± 0.4	34 ± 0	5.0 ± 0.4	5.4 ± 0.3	14.6 ± 0.3	72 ± 2
Kier	Endosperm flour	11.1 ± 0.4	231 ± 6	11.3 ± 0.4	264 ± 6	1.5 ± 0.1	10.6 ± 0.2	35 ± 0	8.2 ± 0.2	6.2 ± 0.1	18.3 ± 0.5	80 ± 0
	A bread	10.0 ± 0.2	217 ± 2	15.9 ± 0.8	253 ± 3	1.7 ± 0.0	10.6 ± 0.4	34 ± 0	5.5 ± 0.1	6.2 ± 0.2	15.7 ± 0.5	74 ± 1
	B bread	9.9 ± 0.5	218 ± 3	15.3 ± 0.4	253 ± 4	2.1 ± 0.2	10.7 ± 0.2	34 ± 1	6.5 ± 0.2	6.5 ± 0.3	16.3 ± 0.3	76 ± 1
	C bread	10.4 ± 0.1	205 ± 5	16.6 ± 0.6	242 ± 4	1.6 ± 0.0	9.8 ± 0.5	32 ± 2	5.2 ± 0.2	5.7 ± 0.2	15.0 ± 0.6	69 ± 4
	D bread	10.8 ± 0.1	237 ± 5	16.5 ± 0.6	274 ± 4	1.9 ± 0.0	11.9 ± 0.5	39 ± 2	6.2 ± 0.2	7.6 ± 0.5	17.9 ± 0.6	84 ± 4
	E bread	10.9 ± 0.1	238 ± 1	18.3 ± 0.7	279 ± 1	2.2 ± 0.1	11.7 ± 0.0	38 ± 1	6.9 ± 0.0	7.6 ± 0.4	17.3 ± 0.1	84 ± 0
Stanko	Endosperm flour	9.7 ± 0.3	217 ± 2	5.0 ± 0.4	240 ± 1	2.3 ± 0.1	8.7 ± 0.3	33 ± 0.1	6.1 ± 0.3	4.5 ± 0.2	17.1 ± 0.1	72 ± 1
	A bread	11.2 ± 0.3	245 ± 5	17.8 ± 0.3	284 ± 4	2.6 ± 0.1	11.2 ± 0.2	37 ± 0.1	5.6 ± 0.1	6.5 ± 0.3	17.3 ± 0.2	80 ± 2
	B bread	10.6 ± 0.3	245 ± 4	17.0 ± 0.8	283 ± 5	2.8 ± 0.1	11.4 ± 0.5	38 ± 0.2	6.2 ± 0.4	7.3 ± 0.3	17.2 ± 0.4	83 ± 1
	C bread	10.7 ± 0.2	235 ± 5	17.9 ± 0.1	273 ± 3	2.4 ± 0.5	11.1 ± 0.3	36 ± 0.3	5.0 ± 0.1	6.6 ± 0.2	16.9 ± 0.1	78 ± 0
	D bread	9.7 ± 0.2	219 ± 4	13.3 ± 0.7	250 ± 6	1.9 ± 0.1	9.6 ± 0.1	33 ± 0.0	4.0 ± 0.0	5.0 ± 0.0	15.6 ± 0.2	69 ± 1
	E bread	11.1 ± 0.2	249 ± 6	15.4 ± 0.8	285 ± 9	2.4 ± 0.2	11.4 ± 0.0	38 ± 0.0	5.5 ± 0.3	6.8 ± 0.4	17.5 ± 0.3	82 ± 3
Legenda (winter wheat)		24 ± 1	845 ± 15	17 ± 2	919 ± 19	5.0 ± 0.6	40 ± 0	117 ± 1	28 ± 1	31 ± 2	56 ± 0	276 ± 3
Raweta (spring wheat)		26 ± 2	927 ± 8	40 ± 1	1025 ± 9	5.7 ± 0.3	52 ± 1	138 ± 2	40 ± 1	46 ± 5	78 ± 0	359 ± 6
White wheat bread		7 ± 0	258 ± 2	11 ± 1	285 ± 3	2.0 ± 0.2	10 ± 0	28 ± 1	5 ± 0	6 ± 0	13 ± 0	64 ± 2
Wheat crisp bread		11 ± 1	514 ± 10	31 ± 0	573 ± 15	3.4 ± 0.1	19 ± 1	55 ± 4	6 ± 0	11 ± 1	25 ± 2	119 ± 9
Rye crisp bread		45 ± 1	1029 ± 5	131 ± 5	1236 ± 6	7.2 ± 0.8	50 ± 2	141 ± 2	23 ± 2	36 ± 2	59 ± 2	317 ± 12
Pumpernickel bread		32 ± 0	806 ± 5	71 ± 1	936 ± 6	7.7 ± 1.0	31 ± 2	98 ± 5	9 ± 1	19 ± 1	27 ± 0	191 ± 13

Data represent mean ± standard deviation.

a Sum of *p*-hydroxybenzoic acid, vanillic acid, caffeic acid, syringic acid, vanillin, *p*-coumaric acid, sinapic acid and ferulic acid.

b TriFA, 8-*O*-4′,5,5′-triFA.

In contrast, the wholemeal breads produced by three-stage sourdough procedures had, in some cases, increased, decreased or the same level of insoluble-bound PAs when compared with that of the wholemeal flour. This can be ascribed to different biochemical characteristics of the native starters and resulting sourdoughs. Among five rye cultivars only Horyzo assured the highest and stable rise in the content of insoluble-bound PAs in the sourdough breads (15, 16 and 15%, respectively for breads C, D and E), irrespective of the first stage length. A gradual decrease in their content was observed in sourdough breads C, D and E made from Kier (17, 13 and 5%, respectively). The sourdough bread obtained from Stanko required the longest time for starter preparation to achieve the highest increase in the concentration of ester-bound fraction (2, 5 and 15%, respectively for breads C, D and E), while that from Amilo after 48-h starter fermentation exhibited only 3% increase. In contrast, in the sourdough breads made from Diament, the amount of insoluble-bound PAs decreased by 1% in bread C and by 8 and 7% in breads D and E. This could be, to some extent, explained by the highest concentration of DiFAs found in the wholemeal obtained from rye cultivar Diament, in comparison to those produced from other rye cultivars, suggesting higher branching degree of cell-wall arabinoxylans.

In the case of bread-making from rye endosperm flour, however, for one rye cultivar Amilo it resulted in substantially increased concentration of insoluble-bound PAs in all types of bread (36, 36, 31, 24 and 26%, respectively for breads A, B, C, D and E). The similar trends with lower increases were found for Stanko, but only in four breads (18, 18, 14, 4 and 18%) and for Diament in three breads (11, 11, 26, 1 and 4%). The endosperm breads made from Horyzo and Kier showed, in most cases, slightly reduced level of this fraction.

The composition of the insoluble-bound fraction present in the wholemeals resembled that in the resulting breads. Ferulic acid, sinapic acid and *p*-coumaric acid accounted for 85–86%, 6–8% and 3–5% of this fraction, respectively. The other minor constituents, such as vanillin, represented about 1% and vanillic acid, syringic acid, caffeic acid and *p*-hydroxybenzoic acid constituted less than 1%. The corresponding fraction in the endosperm flours exhibited higher level of ferulic acid (88–90%) and lower than that of sinapic acid (1–4%). A decreased proportion of ferulic acid (85–88%) and an increased proportion of sinapic acid (4–7%) were observed for insoluble-bound fraction of rye endosperm bread.

The insoluble-bound PAs in the endosperm breads A, B and C were strongly positively linked to the activity levels of *α*-arabinofuranosidase (*r* = 0.95, *r* = 0.96. and *r* = 0.97, *P* < 0.05), *β*-xylosidase (*r* = 0.85, *r* = 0.87 and *r* = 0.82) and feruloyl esterase (*r* = 0.86, *r* = 0.84 and *r* = 0.94, *P* < 0.05). Inversely, in the wholemeal breads these relationships were negative with lower correlation coefficients. Though, a strong correlation was found between the endo-xylanase activity and insoluble-bound fraction for wholemeal breads A and B (*r* = −0.88, *r* = −0.87, *P* < 0.05). It is known that arabinoxylan-hydrolysing enzymes may act synergistically with cinnamoyl esterases, as depolymerisation of long, branched and cross-linked arabinoxylan chains, make them more accessible for hydrolytic action of cinnamoyl esterases. An array of other endogenous enzymes and those secreted by yeast and lactic acid bacteria during sourdough fermentation are present in the dough and, to certain extent, may support their hydrolytic actions. This was evident for bread-making with endosperm flour with much lower concentration of PAs, as positive associations were found between them. In contrast, arabinoxylan-hydrolysing enzymes were inhibited by PAs, occurring at the concentration typical for rye wholemeals. The inhibitory effects of different PAs on activities of *α*-amylase, trypsin and lysozyme have been demonstrated in *in vitro* experiments.^38^ They were related to the blocking of side chains of lysine, the indole ring of tryptophan residues and free thiol groups of cysteine side chains of the enzyme proteins.

The wholemeals obtained from five rye cultivars contained 269–354 µg g^−1^ DM of total DiFAs, including one TriFA, while in the corresponding endosperm flours their level varied from 35 to 80 µg g^−1^ DM (Table3). Their wholemeal values are closely related with those reported previously (241–409 µg g^−1^ DM) by Andreasen *et al.*^8^ The level of DiFAs in wholemeal breads ranged from 252 to 356 µg g^−1^ DM; however, in some cases, it was higher than in the corresponding wholemeal (up to 19% increase). This was more frequent in the endosperm breads where much higher rise in their content was observed (up to 34% increase). One of the explanations for the above is much lower degree of lignification of rye endosperm^26^ with more available cell walls for enzymatic actions. Much like PAs, the DiFAs contents in different types of bread were correlated with feruloyl esterase activity level in the rye endosperm flour (*r* = 0.82, 0.81, 0.88, 0.67 and 0.67, respectively for breads A, B, C, D and E) and in the rye wholemeal (*r* = 0.46, 0.89, 0.82, 0.58 and 0.81).

In several *in vitro* tests, it has been demonstrated that insoluble-bound PAs of wheat grain exhibit significantly higher antioxidant capacity than free and soluble-ester-bound fractions.^39^ Likewise, the *in vitro* assays showed that DiFAs may be more effective inhibitors of lipid peroxidation than ferulic acid.^14^ Both ester-linked PAs and DiFAs present in human diet may contribute to its antioxidant activity *in vivo*, as they can be released from ester linkages by hydrolytic action of intestinal esterases and their monomeric and dimeric forms can be absorbed via the gastro-intestinal tract.^40^

### Bread-making-induced changes in total phenolic acids

**Figure 3:**
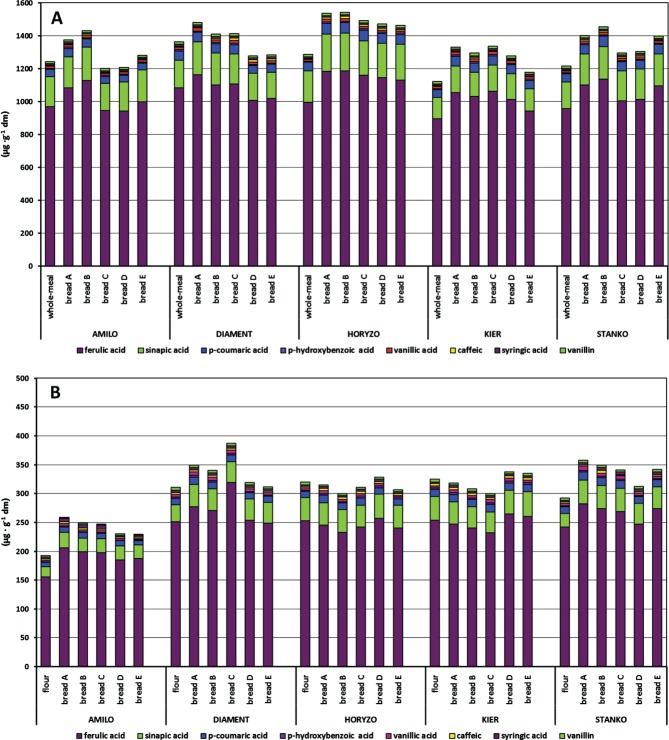
Content and composition of total PAs in (A) rye wholemeals and (B) endosperm flours and resulting breads from five experimental model systems. PAs are arranged from ferulic acid to vanillin from the bottom of each column to the top.

Generally, total PA content was higher in the bread than in the flour (Fig. 3). This was especially evident for rye wholemeal breads A and B, produced by direct methods without and with lactic acid addition. The comparable amounts of total PAs in these breads indicate that both enzymatic and acid hydrolyses were involved in the release of free and soluble-bound PAs. They also contributed to the improved availability of insoluble-bound fraction. The sourdough methodology generally was not as effective as the direct ones, in the terms of total PA content of wholemeal bread. The time of native starter production differently influenced their concentration in the bread C, D and E that was controlled by rye genotype. For endosperm flours and breads made from rye cultivars Horyzo and Kier, the changes in the content of this fraction were negligible, in contrast to those of the remaining samples.

### Multiple linear regression analysis

To point out the most important factors that influenced the level of PAs in rye bread, a linear model for each PA fraction was developed using multiple regression analysis. The adjusted coefficients of determination, standardised beta weights and relative weights of independent variables are shown in Table4. The statistically significant models explained 83 and 85% of the total variation of free PAs in rye wholemeal and endosperm bread, respectively. For soluble-bound (78 and 63%) and insoluble-bound PAs (69 and 75%), the lower values were obtained. The technological factors, such as bread-making methodology and dough/bread pH, were the major contributors to the amount of free PAs in rye bread. The genetic factors, which included the rye genotype and activity levels of enzymes, controlled the contents of soluble-bound and insoluble-bound fractions. Among them, the endoxylanase activity and genotype were the principal determinants of the former fraction, respectively for wholemeal bread and endosperm breads. The insoluble-bound PAs of wholemeal bread were strongly influenced by rye genotype and xylosidase activity, whereas, for those of endosperm bread, the arabinofuranosidase activity and rye genotype were highly related.

**Table 4:** Adjusted coefficients of determination (*R*^2^), standardised beta weights (β^*^) and relative weights (RWs) obtained for independent variables in multiple regression fitting models predicting the contents of free, soluble-bound and insoluble-bound phenolic acids in rye bread

Parameter	Wholemeal bread			Endosperm bread		
	Free	Soluble-bound	Insoluble-bound	Free	Soluble-bound	Insoluble-bound
Coefficient of determination	0.83**	0.78**	0.69**	0.85**	0.63**	0.75**
Standardised beta weights						
Methodology	−0.62*	−0.31*	−0.36*	−0.73*	—	—
Genotype	0.45*	0.48*	0.67*	—	0.54*	0.36*
pH	0.60*	−0.34*	−0.26	0.76*	−0.19	—
Feruloyl esterase	0.14	—	0.19	—	—	—
Endo-xylanase	−0.23	−0.65*	−0.40*	—	0.46*	0.22
Arabinofuranosidase	—	—	—	0.29*	—	0.53*
Xylosidase	−0.40*	−0.40*	−0.65*	—	—	—
Relative weights^a^						
Methodology	32	9	10	44	—	—
Genotype	18	23	35	—	54	28
pH	30	11	5	48	8	—
Feruloyl esterase	2	—	3	—	—	—
Endo-xylanase	4	41	13	—	38	11
Arabinofuranosidase	—	—	—	8	—	61
Xylosidase	14	16	34	—	—	—

Significance levels are:

* *P* < 0.05,

** *P* < 0.01, *n* = 25.

a Relative weights for free, soluble-bound and insoluble-bound phenolic acids.

## Conclusions

The results of this study showed that, during bread-making, free PAs were effectively released from soluble ester-bound and insoluble ester-bound fractions present in the flour by hydrolytic action of cinnamoyl esterases, particularly feruloyl esterase, which was positively related to dough pH. The increased dough acidity hinders hydrolytic action of this enzyme that is partly replaced by acid hydrolysis, as evidenced by the contents of free PAs in the breads obtained by both direct methods, with and without lactic acid addition. However, it was much less efficient than enzymatic de-esterification. Prolonged native starter fermentation, in general, favours the conversion of free forms by sourdough microbiota to their derivatives.

The cell wall-bound PAs and DiFAs, in most cases, become more soluble in resulting bread. This is mostly ascribed to a combined action of feruloyl esterase with many other endogenous enzymes as well as acids added or produced during sourdough fermentation that result in a partial degradation of major dough components. The arabinoxylan-hydrolysing enzymes in rye wholemeal were inhibited by PAs, unlike those in the endosperm flour that could act synergistically with feruloyl esterase during dough preparation. Clearly, the level of phenolics in rye breads produced by sourdough methods was much more dependent on rye cultivar than that in the breads obtained by direct bread-making methods.

The rye cultivars investigated may be categorised into groups that responded differently to the bread-making procedure. Horyzo was distinguished by the highest level of total PAs and DiFAs in the resulting wholemeal breads obtained by both direct and sourdough methods, irrespective of the starter fermentation time. For Amilo, the direct bread-making was the only efficient methodology with respect to maximised concentration of these fractions. Stanko required extended native starter fermentation. In contrast, Diament and Kier needed only short time for starter preparation. This emphasises the importance of rye cultivar in production of the bread with improved bioavailability of PAs.
